# Complete plastome sequence of *Dillenia indica* linn. (Dilleniaceae): an Endangered (EN) species in South China

**DOI:** 10.1080/23802359.2018.1536454

**Published:** 2018-11-25

**Authors:** Xin-Hang Tan, Jian-Hua Wang, Kun-Kun Zhao, Zhi-Xin Zhu, Hua-Feng Wang

**Affiliations:** Hainan Key Laboratory for Sustainable Utilization of Tropical Bioresources, Institute of Tropical Agriculture and Forestry, Hainan University, Haikou, China

**Keywords:** *Dillenia indica* Linn., plastome, phylogeny, genome structure, Dilleniaceae

## Abstract

*Dillenia indica* Linn. is an endangered plant species occurring in southern Guangxi province and Yunnan province of China, which has been grown in gardens as an ornamental plant and is widely used in the medical field. Here, we report and characterize the complete plastid genome sequence of *D. indica* to provide genomic resources useful for promoting its conservation. The complete plastome is 159,266 bp in length and contains the typical structure and gene content of angiosperm plastome, including two inverted repeat (IR) regions of 26,457 bp, a large single-copy (LSC) region of 88,305 bp, and a small single-copy (SSC) region of 18,047 bp. The plastome contains 115 genes, consisting of 81 unique protein-coding genes, 30 unique tRNA genes, and 4 unique rRNA genes (5S rRNA, 4.5S rRNA, 23S rRNA, and 16S rRNA). The overall A/T content in the plastome of *D. indica* is 63.40%. The complete plastome sequence of *D. indica* will provide a useful resource for the conservation genetics of this species as well as for the phylogenetic studies of Dilleniaceae.

*Dillenia indica* Linn. (Dilleniaceae) is an evergreen tree grown in gardens as an ornamental plant (Kumar et al. 2011). It is a rare and Endangered plant of the family Dilleniaceae (Qin et al. 2017) and widely distributed in valleys and streamsides in southern Guangxi province and Yunnan province of China (Zhang and Klaus 2007). It is used for relieving abdominal pain and its fruit shows laxative properties (Kirtikar and Basu 2001). *Dillenia indica* was sampled from Baoting country in Hainan province of China (109.701°E, 18.642°N). A voucher specimen (Wang et al., B117) was deposited in the Herbarium of the Institute of Tropical Agriculture and Forestry (HUTB), Hainan University, Haikou, China.

Around 6 Gb clean data were assembled against the plastome of *Ampelopsis glandulosa* (KT831767.1) (Raman and Park 2016) using MITO bim v1.8 (Hahn et al. 2013). The plastome was annotated using Geneious R8.0.2 (Biomatters Ltd., Auckland, New Zealand) against the plastome of *Vitis aestivalis* (KT997470.1). The annotation was corrected with DOGMA (Wyman et al. 2004).

The plastome of *D. indica i*s found to possess a total length 159,266 bp with the typical quadripartite structure of angiosperms, containing two inverted repeats (IRs) of 26,457 bp, a large single-copy (LSC) region of 88,305 bp, and a small single-copy (SSC) region of 18,047 bp. The plastome contains 115 genes, consisting of 81 unique protein-coding genes (seven of which are duplicated in the IR: *rps12, rps7, ndhB, ycf15, ycf2, rpl23*, and *rpl2*), 30 unique tRNA genes (seven of which are duplicated in the IR: *trnN-GUU, trnR-ACG, trnA-UGC, trnI-GAU, trnV-GAC, trnL-CAA* and *trnI-CAU*), and 4 unique rRNA genes (5S rRNA, 4.5S rRNA, 23S rRNA, and 16S rRNA). Among these genes, 4 pseudogenes (*ndhK*, *accD*, *rps19*, and *ycf1*), 15 genes (*trnK-UUU, trnG-GCC, trnL-UAA, trnV-UAC, trnI-GAU, trnA-UGC, rps16, atpF, rpoC1, petB, petD, rpl16, rpl2, ndhB*, and *ndhA*) possessed a single intron and three genes (*ycf3, clpP,* and *rps12*) had two introns. The gene *rps12* was found to be trans-spliced, as is typical of angiosperms. The overall A/T content in the plastome of *D. indica* is 63.40%, for which the corresponding value of the LSC, SSC and IR region were 65.80%, 69.80% and 57.20%, respectively.

We used RAxML (Stamatakis 2006) with 1000 bootstraps under the GTRGAMMAI substitution model to reconstruct a maximum likelihood (ML) phylogeny of 5 published complete plastomes of Saxifragales, 2 published complete plastomes of Caryophyllales, 2 published complete plastomes of Santalales, 2 published complete plastomes of Buxales, 2 published complete plastomes of Trochodendrales, using *Sabia yunnanensis* (Sabiaceae, Proteales) and *Macadamia ternifolia* (Proteaceae, Proteales) as outgroups. The phylogenetic analysis indicated that *D. indica* is closer to Caryophyllales than other orders in this study (Figure 1). Most nodes in the plastome ML trees were strongly supported. The complete plastome sequence of *D. indica* will provide a useful resource for the conservation genetics of this species as well as for the phylogenetic studies of Dilleniaceae.

**Figure 1.**
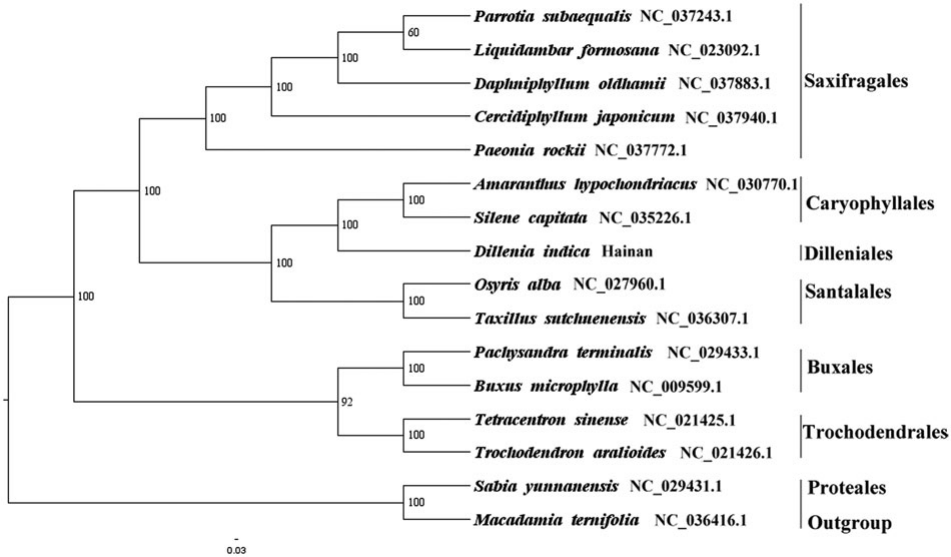
The best ML phylogeny recovered from 16 complete plastome sequences by RAxML. Accession numbers: *Dillenia indica* Linn. (This study, GenBank Accession number: MH708162), *Parrotia subaequalis* NC_037243.1, *Liquidambar formosana* NC_023092.1, *Daphniphyllum oldhamii* NC_037883.1, *Cercidiphyllum japonicum* NC_037940.1, *Paeonia rockii* NC_037772.1*, Amaranthus hypochondriacus* NC_030770.1, *Silene capitata* NC_035226.1, *Osyris alba* NC_027960.1, *Taxillus sutchuenensis* NC_036307.1, *Pachysandra terminalis* NC_029433.1, *Buxus microphylla* NC_009599.1, *Tetracentron sinense* NC_021425.1, *Trochodendron aralioides* NC_021426.1; outgroups: *Sabia yunnanensis* NC_029431.1, *Macadamia ternifolia* NC_036416.1.
